# On Diuretics in the Treatment of Malarious or Periodical Fevers

**Published:** 1865-05

**Authors:** N. Wright

**Affiliations:** Chatham, Ill.


### ARTICLE XVII.

### ON DIURETICS IN THE TREATMENT OF MALARIOUS OR PERIODICAL FEVERS. Read to the Meeting of the Illinois State Medical Society, May 3, 1865.

By N. WRIGHT, M.D., Chatham, Ill.

In the able report on Practical Medicine, read before the Society at its last meeting, it was very properly stated that one of the ways in which Practical Medicine might be improved, was by the new application of old remedies, more or less known; and, by blessing of nature, having been constituted an old fogy, "seeking for the old paths, and loving the old ways," I hope it will not be considered obtrusive in me to introduce one of my old friends and valued assistants.

The physician who has practiced for any considerable time in one of our alluvial districts, has met with numerous cases of malarious origin, in their various protean forms, that defiantly refuse to yield to the usual treatment with anti-periodics. We are often called upon, again and again, to treat the same patient for a return of the "chills and fever," as they term it, until the patient, if not the physician, loses all faith in quinine, the Sampson in malarious diseases, to accomplish a cure, and restore the patient to health. The unbelief of the patient often leads him to the alternative of wearing out the disease, rather than take the medicine which his experience has demonstrated to him only checks, or gives but temporary respite from the ague, without a cure of the disease. These cases of chronic malarial poisoning are so frequent that it seems almost supererogation to enumerate their appearances. The sallow, waxen-look of the countenance; the yellow look of the conjunctiva; the torpor of the intellectual faculties; the general anæmia, and dropsy-like appearance of the patient; the hepatic, gastric, and renal disturbances, all speak to the observing physician the tale of chronic malarial poisoning.

I do not here propose to enter into any discussion as to the exact nature of *the* poison—whether it be a certain gas or gaseous compound, as affirmed by some; or by certain electrical changes in the atmosphere, as believed by others; or by hosts of spores of cryptogamic fungi in circulation through the atmosphere, as is affirmed by another class of medical men—sufficient for me and my purpose at this time, is the admitted fact, that whatever the generic poison or *casus mali* may be, its first perceptible effects are upon the nervous system, and especially upon the organic or ganglionic nerves that preside over the secretive functions. No secretion escapes the influence of the poison—the hair, skin, kidneys, all feel the polluting touch. Their secretions are changed both in quantity and quality, and as the blood is the material from which all the various secretions are eliminated, it must also be the *point d'appui* upon the hydra-headed monster, malarial poisoning. If the general appearance of this class of patients did not sufficiently indicate the changes wrought by the poison in the blood, the microscope reveals the fact of an abnormal preponderance of white, and a corresponding diminution of red corpuscles in the circulation of the blood itself. There is no doubt, in my mind, that the changed condition of the blood, and the consequent abnormal secretions, or rather almost entire suspension of secretions of all kinds, are the powerful agents, together with the original cause, which tend in a great measure to keep up a morbid action. To restore the various secretions to their normal condition, is, unquestionably, the first object to be attained in the treatment of this class of diseases. Small doses of mercury do much to remove the partial congestions, but this is not all that is required, the effete matter, unremoved from the blood, cannot so readily (if, indeed, they can be at all, which is very questionable in my mind) be removed by the liver as by the kidneys—the sewers of the blood. The carbonized matters are the especial province of the liver to remove from the blood, but there is still a large amount of nitrogenized, worn-out, broken-down, deleterious matter, that must be eliminated from the system before we can cure the patient. In this class of diseases, the urine is usually, if not always, much diminished in quantity and quality, and the solid matters in solution are more diminished than even the quantity. Three to four hundred grains of solids are the usual amounts deposited, while from six to seven hundred ought to be removed in the normal state. Now, if we can introduce some therapeutic agent that will enter the blood and capillary network of the body, and thus aid the metamorphosis and excretions from the blood of the unhealthy elements there contained, and remove them from the system, we are working in a rational and sure way to cure our patients. The agent that I would introduce is acetate of potash, freely diluted with water, about ʒj to the pint, two or three times a day. I have tried it repeatedly, and generally with success.

I would not dispense with quinine, to check the paroxysm, nor with some of the forms of mercury, to remove the partial congestion; but they do not remove the disease, nor its products in the changed various secretions. This is done by acetate of potash, through the secretion of the kidneys.

Having treated ague with mercurials, quinine, and tonics alone, five years, with not complete satisfaction to myself (to say nothing of my patients), permit me to say, that the success for the past two years, under this plan that I have so imperfectly given you the outlines of, has been such as to justify me in asking you to give it a fair trial under favorable circumstances.

